# Zinc Deficiency Interacts with Intestinal/Urogenital Parasites in the Pathway to Anemia in Preschool Children, Bengo–Angola

**DOI:** 10.3390/nu14071392

**Published:** 2022-03-27

**Authors:** Cláudia Fançony, Ânia Soares, João Lavinha, Miguel Brito

**Affiliations:** 1 Centro de Investigação em Saúde de Angola (CISA), Caxito, Estação Central de Luanda, Apartado IV n °5547, Luanda 5547, Angola; ania.soares@cisacaxito.org (Â.S.); miguel.brito@estesl.ipl.pt (M.B.); 2 Departamento de Genética Humana, Avenida Padre Cruz, Instituto Nacional de Saúde Dr. Ricardo Jorge, 1649-016 Lisboa, Portugal; joao.lavinha@hotmail.com; 3 Health and Technology Research Center, Escola Superior de Tecnologia da Saúde de Lisboa, Instituto Politécnico de Lisboa, Av. Dom João II Lote 4.69 01, 1990-096 Lisboa, Portugal

**Keywords:** zinc deficiency, anemia, infections, inflammation

## Abstract

In host organisms with normal micronutrient status, nutritional immunity is a strongly regulated response aiming at decreasing the progression and severity of infections. Zinc deficiency may disturb this balance, impairing immune responses to infections, which may indirectly increase infection-related anemia. Since zinc deficiency may associate directly with anemia, the role of infections is often overlooked. Herein, we investigated the participation of infections (or inflammation) in the causal pathway between zinc deficiency and anemia. This transversal study, conducted in 2015 in Bengo-Angola, enrolled 852 under-3-year-old children. Logistic regression models were used to investigate interaction and mediation effects, and significance was confirmed by the Sobel test. In sum, 6.8% of children had zinc deficiency, 45.9% had anemia, and 15.6% had at least one intestinal/urogenital parasite. Furthermore, we found (1) no evidence that inflammation mediates or interacts with zinc deficiency to cause anemia, and (2) zinc deficiency interacts with infections, significantly increasing the odds of anemia (OR: 13.26, *p* = 0.022). This interaction was stronger among children with iron deficiency anemia (OR: 46.66, *p* = 0.003). Our results suggest that zinc deficiency may impair the immune response to infections and/or that intestinal parasites could have developed mechanisms to avoid zinc-limited environments. Further studies are needed to corroborate these suggestions.

## 1. Introduction

Nutritional immunity is the process by which the host manages the availability of micronutrients (such as zinc and iron) in an attempt to limit the progression and severity of infections [1,2]. In the case of zinc, the main strategies employed are mineral deprivation, intoxication, or sequestration [3]. Furthermore, zinc was reported to both downregulate the Th2 response and to upregulate the Th1 response, acting as a signaling molecule [4]. In all these cases, the strategy employed is pathogen-dependent. For instance, while zinc sequestration was reported to negatively influence *Escherichia coli*, *Staphylococcus aureus*, and *Histoplasma capsulatum*, zinc intoxication was reported to eliminate *Mycobacterium tuberculosis* [3,5,6,7,8]. On the other hand, the distinctive characteristics of helminths (such as their multicellularity) leads to considerable heterogenic immune responses, for whom the type 2 immunity plays an important role [4,9,10,11].

In a host with normal zinc levels, zinc sequestration is a strongly regulated response that aims at starving the invading pathogens, and it may lead to transient lowered zinc levels associated with inflammation [12]. However, deficiencies or excess of this mineral was reported to alter the susceptibility to intestinal parasites [4,10,11]. Furthermore, while acute zinc deficiency may decrease the innate and adaptive immunity response, chronic deficiency may increase the production of inflammatory cytokines, negatively influencing the outcome of inflammatory diseases [13]. On the other hand, while zinc supplementation was reported to lead to a faster resolution of *Giardia*, it can also increase the prevalence of *A*. *lumbricoides* and prolong the duration of *E. histolytica* infections [4]. Nevertheless, in general, zinc deficiency was associated with thymic atrophy, decreased phagocytosis, oxidative burst, chemotaxis, delayed wound healing, and increased susceptibility to infections, while zinc overload was reported to be associated with direct activation of monocytes, increased B cell apoptosis, suppressed T cell functions, and severe pro-inflammatory action during sepsis [3,14].

Since there are no large stores to mobilize zinc, a regular intake of that trace mineral is required. Thus, zinc deficiency is largely related to inadequate intake or absorption of zinc from the diet [13,15,16,17,18]. At the gastrointestinal tract, intestinal parasites may be responsible for this inadequate absorption, consequently leading to malnutrition (undernutrition and/or micronutrient deficiencies) and anemia, through processes of inflammation, malabsorption, chronic blood loss, anorexia, or hemolysis [19,20]. In settings where adequate water and sanitation systems are not in place, frequent exposure to gastrointestinal pathogens occurs and high rates of intestinal parasites and diarrhea may increase zinc intestinal excretion [21]. For instance, children with giardiasis were reported to have lowered zinc absorption, leading to zinc deficiency [13,14]. Since enteric nematodes, cestodes, trematodes, and protozoans often occur sympatrically in endemic areas, polyparasitism has been suggested to have a synergistic effect in exacerbating detrimental health outcomes in infected individuals [19,22,23]. In addition, the intensity of infection and the type of coinfection determine the extension of the negative impact of those processes in the host [19,24].

Despite the existence of plausible mechanisms for the joint participation of zinc deficiency and infections (and infection-related inflammation) in the causal pathway to anemia, there are limited in vivo data documenting these associations. Herein, we aimed to investigate if the causal pathway from zinc deficiency to anemia is influenced (through mediation or interaction) by infections or inflammation. Specifically, we aimed at testing if infections or inflammation directly influence the occurrence of anemia (hypothesis 1), partially mediate the effect of zinc deficiency in the occurrence of anemia (hypothesis 2), or interact with zinc deficiency in its association with anemia (hypothesis 3).

## 2. Material and Methods

### 2.1. Study Design and Population

The present study is nested into a 12-month follow-up effectiveness trial, investigating the effect of nutrition and WASH/malaria educational community-based interventions in reducing anemia in preschool children from Bengo, Angola [25]. The study protocol was approved by the Ethics Committee of the Ministry of Health of the Republic of Angola (10 June 2015) [26]. The data presented here were collected at the baseline of that major study. In brief, it occurred in seven hamlets with functional health posts selected from CISA’s study area [27,28]. The hamlets were selected by convenience, based on the presence of a functional peripheric health unit. After the explanation of the study and a verbal acceptance to participate was manifested, the field technician delivered a participant information form and stool and urine containers to eligible families and instructed them to be present at the health post for evaluation. From all recruited families, 948 under-3-year-old children attended the evaluation day at the health posts, and informed consent was signed (by the caretaker) for each one. Then, children were evaluated. Only children with evaluable biochemical results for zinc pathologic levels were included here.

### 2.2. Sample Collection

The parasitological analysis comprised the diagnosis of *P. falciparum* malaria performed using a rapid diagnostic test *SD BIOLINE* Malaria Ag *P.f*/*P.v^®^* (Standard Diagnostics, Inc., Yongin, Korea), according to the manufacturer’s instructions. Diagnosis of intestinal parasites was performed through fecal smear and centrifugal sedimentation using the Kato–Katz technique and Parasitrap^®^ kits (Biosepar GmbH, Simbach am Inn, Germany). Diagnosis of urogenital schistosomiasis was performed by urine filtration, using Whatman^®^ Nuclepore™ membranes (Merck KGaA, Darmstadt, Germany) [29,30,31]. The biochemical analysis included determining blood levels of hemoglobin by immunochromatography using an Hemocue^®^ Hb 301 System (Angelholm, Sweden), serum levels of C-reactive protein (CRP), ferritin, and zinc, respectively by turbidimetry, immunoturbidimetry, and colorimetry (using an automated autoanalyzer (BT1500) and CRP turbidimetric latex ^®^, Ferritin^®^ and Zinc^®^ kits from Quimica Clínica Aplicada S.A (Tarragona, Spain).

### 2.3. Statistical Analysis

The biochemical diagnostic thresholds were defined as follows: anemia—hemoglobin (Hb) < 11.0 g/dL [18,27,28]; inflammation—CRP > 5 mg/L; iron deficiency—serum ferritin < 12 μg/L (without inflammation) or <30 μg/L (with inflammation) [29] and zinc status—Zn^2+^ < 70.0 μg/dL (deficiency); Zn^2+^ > 150 μg/dL (overload) [30]. The prevalence´s of helminths and protozoa were determined as the proportion between infected children and those delivering the corresponding sample. Children were considered to have diarrhea if caretakers reported that the children had 3 or more aqueous dejections per day in the last 2 weeks.

In this study, 95% confidence intervals (95% CIs) were estimated for the frequencies. Crude multinomial models were fitted for each independent variable, taking children with normal zinc serum levels as the reference category for the dependent variable. Logistic regression models were used to estimate mediation and interaction effects.

### 2.4. Estimation of Mediation 

Mediation was considered to exist if: (1) the independent variable influenced significantly the mediating variable, (2) the independent variable influenced significantly the dependent variable, and (3) the addition of the mediating variable to the model altered significantly the effect of the independent variable in the dependent one (as described in Table 1) [32,33]. In addition to this method, the Sobel test was used to determine the significance of the mediation effect, as proposed by Baron and Kenny (1986).

### 2.5. Estimation of Interaction

Interaction is reported to occur when the effect of an explicative variable on the occurrence of anemia is different across the strata of another explicative variable [34,35]. Here, an interaction was considered to exist if the combined effect of both variables were larger or smaller than the individual effects of A and B in the same model, and statistical significance (*p* < 0.05) was observed (described in Table 2).

## 3. Results and Discussion

The main study included 948 children aged up to 36 months, 54.9% aged 6 to 23 months (mean = 16.8 months, SD = 10.1), and 51.6% males. Among those enrolled, 852 had evaluable data for zinc serum levels. From those, 45.9% (391/851) had anemia, 6.8% (58/852) were found to have zinc deficiency, and 15.6% (117/752) had at least one intestinal or urogenital parasite (mainly *Giardia lamblia* (7.8%, 55/702), *Schistosoma haematobium* (6.8%, 35/513), and *Ascaris lumbricoides* (3.9%, 28/714) (see Table 3). This high frequency of anemia and infections was already reported in this study area [36]. However, the anemia prevalence observed here was lower than the one reported for the municipality in a 2010 representative survey [36].

In the present study, children with zinc deficiency were found to have higher odds of having anemia (OR: 2.0, *p* = 0.013) (see Table 3). In fact, zinc was already described to be a predictor of hemoglobin levels and consequently associated with the occurrence of anemia [37,38,39]. Several aspects were highlighted in the literature, indicating that zinc concentration could affect the absorption of iron and lead to Iron Deficiency Anemia (IDA), and that inflammation could act as a regulatory factor in the association between zinc, hemoglobin, and anemia. On the other hand, children with zinc deficiency had 1.6 more chances of having at least one intestinal/urogenital parasite; however, this association was not statistically significant. Zinc deficiency was reported to potentially lead to immune dysfunctions that consequently worsen responses toward parasites, and the lack statistical significance may be attributed to numeric problems rather than biologic significance [15,16,38,39,40,41,42,43].

### 3.1. Could Zinc Levels and Infections Be Contributing Jointly to Anemia?

In this study, none of the studied intestinal/urogenital parasites were found to occur differentially among zinc status groups, neither alone nor in mixed infections (see Table 4). However, children with either low or high zinc levels were less likely to be infected with *P. falciparum* malaria than children with normal zinc values (association that was statistically significant only for zinc overload). Thus, considering that zinc deficiency was reported to lead to immune dysfunctions that consequently worsen responses toward parasites, we hypothesize that zinc could be mediating or interacting directly with parasites to cause anemia (without the influence of inflammation) [42,43]. Diarrhea was used as a proxy for other enteric parasites. In the present study, no evidence of a mediation relationship were found. However, the condition of having at least one intestinal and/or urogenital parasites was found to interact with zinc deficiency in causing anemia. The association was found to increase significantly the odds of IDA (OR: 46.66, *p* = 0.003, data not shown).

Within a host with normal zinc values, it would be expected that zinc deficiency would result in lower availability of this mineral for helminths, and as a consequence, their survival would decrease, as would also the infectious-related anemia. For instance, induced zinc deficiency was reported to reduce fertility and disrupt oogenesis in Caenorhabditis elegans [44]. Here, parasites have a higher effect on anemia when they are in the presence of zinc deficiency (than when they are in the absence of zinc deficiency). This fact could be highlighting that either zinc deficiency does impair immune response to infections and/or helminths may have developed mechanisms to avoid the consequences of a zinc-limited environment [45,46]. Results from Lee et al. 2019 describe that helminth infections (or microbial communities favored by them) may induce increased zinc levels directly and that either zinc or iron levels were associated with alterations in helminth infection status (independently of their intake) [45]. Other authors have also suggested the existence of a complex interplay between the host nutritional state, intestinal parasites, and immune responses, which is further complicated by the gut microbiota [45,47].

In addition, the association with iron is in accordance with reports mentioning that the body also responds to pathogens by decreasing iron availability, further removing iron from circulation and storing it as ferritin [48]. This fact was reported to be achieved through increased hepcidin, whose production is in turn influenced by pro-inflammatory cytokines [42,49].

### 3.2. Could Zinc Levels and Inflammation Be Contributing Jointly to Anemia?

In general, non-malarial parasites could cause anemia indirectly by leading to blunted intestinal villi, chronic blood loss, hemolysis, and/or inflammation, while malaria was reported to directly cause anemia by hemolysis but also indirectly by inflammation [19,20,50]. In a previous study published by Fançony et al., it has been shown that whereas non-malarial inflammation was reported to occur more frequently in children with IDA than in children with normal hemoglobin (OR: 2.4, *p* < 0.001), malarial inflammation showed no differentiated distribution between IDA, non-IDA, and non-anemic groups when all age groups were considered [51]. Considering reports describing that during inflammatory events, the concentrations of plasmatic zinc is decreased, thus withholding it from the pathogen and that this nutritional immunity could be contributing to anemia, we performed additional analysis to understand if zinc pathologic levels could be involved in those associations [16,42]. We found that although total inflammation had no statistically significant association with pathological zinc levels, children presenting zinc deficiency had 1.9 more chances of having non-malarial inflammation than children with normal levels of zinc, possibly corroborating the participation of zinc in the nutritional immunity to pathogens other than malaria.

Considering this, the second biological plausibility being tested here investigated the possibility that parasites and/or other pathological conditions could be causing inflammation, which could be mediating or interacting with zinc deficiency in the causal pathway to anemia. To further investigate if this pathway could have two levels, i.e., if inflammation was in turn related to parasites, having diarrhea, or at least one intestinal or urogenital parasites, we also considered multiple associations in our mediating or interacting models. In the mediation model (postulating inflammation as a mediator variable), the effect of zinc deficiency on anemia remained similar to the direct effect (OR: 1.93, *p* = 0.022), indicating that the inflammation may not be mediating that relationship. This is also confirmed by the Sobel test (*p* > 0.05). This fact was also observed when we removed children with IDA from the analysis (OR: 2.84, *p* = 0.001 to direct effect and OR: 2.80, *p* = 0.001 for indirect effect) or non-malarial inflammation (OR: 1.38, *p* = 0.439 to direct effect and OR: 1.43, *p* = 0.387 for indirect effect). Furthermore, we also have not found significant evidence of the interaction between zinc deficiency and inflammation in the causal pathway to anemia, as the odds of the combined effect (OR: 1.86, 95% CI: 0.590–5.872, *p* = 0.290) were not significantly different from their effect on anemia. Similar results were observed when the interaction was investigated only to non-IDA children (OR: 1.787, 95% CI: 0.528–6.049, *p* = 0.351). When diarrhea and having at least one intestinal/urogenital parasite were included in the mediation or interaction models of zinc deficiency, inflammation, and anemia, the effects were non-statistically significant. For instance, the odds ratio for multi-mediation between zinc deficiency, inflammation, and diarrhea was 1.80 (95% CI: 1.02–3.18, *p* = 0.042), similar to the odds ratio for the direct effect (OR: 1.97, 95% CI: 1.12–3.45, *p* = 0.018), and despite being higher than the odds ratio of the direct effect, no statistical significance was observed for the odds ratio of the respective multi-interaction model (OR: 1.48, 95% CI: 0.786–2.801, *p* = 0.223 for the direct effect and OR: 2.49 95% CI: 0.58–10.798, *p* = 0.221 for the indirect effect). On the other hand, the odds for the multi-interaction model were 5.44 (95% CI: 0.579–51.138, *p* = 0.138), which were also higher than the direct effect (OR: 1.81, 95% CI: 0.95–3.48, *p* = 0.072) but not significant. In summary, no relevant results were observed regarding the mentioned effects. Nevertheless, inflammation is associated with anemia, either in children with concomitant *P. falciparum* infection or not, and thus should be further investigated.

## 4. Conclusions

In this study, we found that high zinc levels were found to protect against malaria, while zinc deficiency increased the chances of malaria-unrelated inflammation. Furthermore, parasites were found to interact with zinc deficiency, significantly increasing the odds of anemia, especially IDA. No other single, mediation, or interaction models studied here had statistical significance. These results may be supportive of the fact that zinc deficiency impairs the host immune response to intestinal/urogenital infections and/or that these parasites may have developed mechanisms to avoid zinc-limited environments. Nevertheless, further studies are needed to confirm this. Plus, these results should be interpreted with care, as multi-causal pathways are complex and other types of associations were not explored, such as suppression or moderated mediation. As far as we know, there are no similar studies published on the Angolan pediatric population, highlighting the joint contribution of zinc deficiency and intestinal/urogenital infections to the occurrence of anemia.

## Figures and Tables

**Table 1:** Mediation effects being tested.

	Models	Effect Being Tested
Does inflammation (or malarial inflammation) mediate the effect of zinc deficiency in anemia?		
Model 1: Bivariate regression	Anemia = B0 + B1 zinc deficiency + e1	Direct effect of zinc deficiency on anemia
Model 2: Bivariate regression	Inflammation = B0 + B2 zinc deficiency + e2	Direct effect of zinc deficiency on inflammation
Model 3: Multiple regression	Anemia = B0 + B3 zinc deficiency + B2 inflammation + e3	Joint effect of zinc deficiency and inflammation on anemia
Does having at least one intestinal/urogenital parasite mediate the effect of zinc deficiency in anemia?		
Model 4: Bivariate regression	Anemia = B0 + B1 zinc deficiency + e1	Direct effect of zinc deficiency on anemia
Model 5: Bivariate regression	Having at least one intestinal/urogenital = B0 + B2 zinc deficiency + e2	Direct effect of zinc deficiency on having at least one intestinal/urogenital
Model 6: Multiple regression	Anemia = B0 + B3 zinc deficiency + B2 having at least one intestinal/urogenital + e3	Joint effect of zinc deficiency and having at least one intestinal/urogenital on anemia

Variables: Anemia (0 = no, 1 = yes), zinc deficiency (0 = no, 1 = yes), inflammation (0 = no, 1 = yes).

**Table 2:** Interaction effects being tested.

	Models	Effect Being Tested
Does zinc deficiency interact with inflammation to cause anemia?		
Model 7: Bivariate regression	Anemia = B0 + B1 zinc deficiency + e1	Direct effect of zinc deficiency on anemia
Model 8: Bivariate regression	Anemia = B0 + B2 inflammation + e2	Direct effect of inflammation on anemia
Model 9: Multiple regression	Anemia = B0 + B3 zinc deficiency + B2 Inflammation (or malarial inflammation) + B3 zinc deficiency * Inflammation (or malarial inflammation) + e3	Joint effect of zinc deficiency and inflammation on anemia
Does zinc deficiency interact with having at least one intestinal/urogenital parasite to cause anemia?		
Model 10: Bivariate regression	Anemia = B0 + B1 zinc deficiency + e1	Direct effect of zinc deficiency on anemia
Model 11: Bivariate regression	Anemia = B0 + B2 having at least one intestinal/urogenital parasite + e2	Direct effect of having at least one intestinal/urogenital parasite on anemia
Model 12: Multiple regression	Anemia = B0 + B3 zinc deficiency + B2 having at least one intestinal/urogenital parasite + B3 zinc deficiency having at least one intestinal/urogenital parasite + e3	Joint effect of zinc deficiency and having at least one intestinal/urogenital on anemia

Variables: Anemia (0 = no, 1 = yes), zinc deficiency (0 = no, 1 = yes), having at least one intestinal/urogenital parasite (0 = no, 1 = yes). * Term that symbolizes the interaction between independent variables.

**Table 3:** Associations between pathologic zinc levels and explicative study variables.

	Total Population	Frequency among Zinc Level Groups			Association with Zinc Levels *				
		Normal	Low	High	Normal	Low		High	
	% (*n*/N)	% (*n*/N)	% (*n*/N)	% (*n*/N)		OR (95% CI)	*p*-Value	OR (95% CI)	*p*-Value
Anemia									
No	54.1 (460/851)	53.5 (336/628)	36.2 (21/58)	62.4 (103/165)	Ref	Ref	Ref	Ref	Ref
Yes	45.9 (391/851)	46.5 (292/628)	63.8 (37/58)	37.6 (62/165)		2.0 (1.2–3.5)	0.013	0.7 (0.5–1)	0.041
P. falciparum									
No	94.7 (805/850)	93.3 (585/627)	98.3 (57/58)	98.8 (163/165)	Ref	Ref	Ref	Ref	Ref
Yes	5.3 (45/850)	6.7 (42/627)	1.7 (1/58)	1.2 (2/165)		0.2 (0–1.8)	0.168	0.2 (0–0.7)	0.015
At least one intestinal or urogenital parasite									
No	83.5 (628/752)	85.3 (464/544)	78.4 (40/58)	82.7 (124/150)	Ref	Ref	Ref	Ref	Ref
Yes	15.6 (117/752)	14.7 (80/544)	21.6 (11/58)	17.3 (26/150)		1.6 (0.8–3.2)	0.196	1.2 (0.7–2)	0.429
Inflammation									
No	54.8 (463/845)	55.1 (343/623)	41.4 (24/58)	58.5 (96/164)	Ref	Ref	Ref	Ref	Ref
Malarial **	3.3 (28/845)	4.0 (25/623)	56.9 (33/58)	1.2 (2/164)		0.6 (0.07–4.4)	0.591	0.3 (0.07–1.2)	0.092
Non-malarial ***	41.9 (354/845)	40.9 (255/623)	1.7 (1/58)	40.2 (66/164)		1.9 (1.1–3.2)	0.028	0.9 (0.7–1.3)	0.664
Diarrhea									
No	59 (497/843)	59.5 (370/622)	71.9 (41/57)	52.4 (86/164)	Ref	Ref	Ref	Ref	Ref
Yes	41 (346/843)	40.5 (252/622)	28.1 (16/57)	47.6 (78/164)		0.6 (0.3–1)	0.069	1.3 (0.9–1.9)	0.104

* Crude multinomial models. ** Malarial inflammation: occurrence of both inflammation and malaria. *** Non-malarial inflammation: occurrence of inflammation in the absence of malaria.

**Table 4:** Main mediation and interaction results for the hypothesis tested in this study.

Model_Effect	Independent Variables	Dependent Variable	OR (IC95%)	*p*-Value
Does inflammation (or malarial inflammation) mediate the effect of zinc deficiency on anemia?				
1_Med.	Zinc deficiency	Anemia	2.03 (1.16, 3.54)	0.013
2_Med.	Zinc deficiency	Inflammation	1.70 (0.99, 2.94)	0.055
3_Med.	Zinc deficiency	Anemia	1.93 (1.10, 3.38)	0.022 *
	Inflammation		1.57 (1.16, 2.12)	0.004
Does having at least one intestinal/urogenital parasite mediate the effect of zinc deficiency on anemia?				
4_Med.	Zinc deficiency	Anemia	2.30 (1.26, 4.22)	0.007
5_Med.	Zinc deficiency	Having at least one parasite #	1.60 (0.79, 3.24)	0.196
6_Med.	Zinc deficiency	Anemia	2.39 (1.30, 4.40)	0.005 *
	Having at least one parasite #		0.63 (0.40, 1.00)	0.051
Does zinc deficiency interact with inflammation to cause anemia?				
7_Int.	Zinc deficiency	Anemia	1.38 (0.60, 3.16)	0.444
8_Int.	Inflammation	Anemia	1.49 (1.09, 2.05)	0.013
9_Int.	Zinc deficiency * Inflammation	Anemia	1.86 (0.59, 5.87)	0.290
Does zinc deficiency interact with having at least one intestinal/urogenital parasite to cause anemia?				
10_Int.	Zinc deficiency	Anemia	1.57 (0.81, 3.02)	0.182
11_Int.	Having at least one parasite #	Anemia	0.50 (0.30, 0.83)	0.007
12_Int.	Zinc deficiency * Having at least one parasite #	Anemia	13.26 (1.46, 120.72)	0.022

* *p*-value (Sobel Test) < 0.05. # Having at least one intestinal/urogenital parasite (*A. lumbricoides*, *T. trichiura*, *G. lamblia*, *H. nana*, *E. hystolitica*, *S. mansoni* and *S. haematobium*). Med.—Mediation. Int.—Interaction.

## Data Availability

The data supporting the results reported here can be made available upon request.
